# Correlation Analysis of Blood Glucose Level with Inflammatory Response and Immune Indicators in Patients with Sepsis

**DOI:** 10.1155/2022/8779061

**Published:** 2022-05-26

**Authors:** Qi Wei, Jinglin Zhao, Hao Wang, Cuicui Liu, Caihong Hu, Chao Zhao, Qingchun Dai, Zhi Hui, Rui Wang

**Affiliations:** ^1^Department of Critical-Care Medicine, Cangzhou Central Hospital, Cangzhou, China; ^2^Department of Pharmacology, Cangzhou Medical College, Cangzhou, China; ^3^Department of Imaging, Cangzhou Medical College, Cangzhou, China

## Abstract

**Objective:**

To analyze the correlation of blood glucose level with inflammatory response and immune indicators in patients with sepsis.

**Methods:**

Between February 2019 and February 2021, 30 sepsis patients and 30 sepsis patients complicated with diabetes mellitus admitted to our hospital were recruited and assigned to either the experimental group (sepsis patients) or the observation group (sepsis patients with diabetes mellitus). Another 30 healthy subjects in the same period were included as the control group. The levels of IL-6, TNF-*α*, IL-1*β*, CD4+, and CD8+ in the three groups of patients were compared to analyze the correlation of blood glucose levels with inflammatory response and immune indicators in patients with sepsis. The difference of counting data was analyzed using the chi-square test, and the difference of measurement data was analyzed using the *t*-test.

**Results:**

The control group showed the lowest levels of IL-6 at 14.32 ± 4.98 pg/ml, followed by 18.33 ± 3.27 pg/ml in the experimental group and then 22.64 ± 5.16 pg/ml in the observation group (*P* < 0.05). The levels of other inflammatory factors including TNF-*α* and IL-1*β* were the lowest in the control group, followed by the experimental group, and then the observation group (*P* < 0.05). The lowest immune function indicator CD4+ and CD8+ levels were found in the observation group, followed by the experimental group, and then the control group (*P* < 0.05). The blood glucose level of patients with sepsis was positively correlated with the levels of IL-6, TNF-*α*, and IL-1*β* and was negatively correlated with the levels of CD4+/CD8+. The higher the blood glucose, the lower the number of immune cells.

**Conclusion:**

The blood glucose level of patients with sepsis is positively correlated with inflammatory response and negatively with immune indicators. An increased blood sugar level is associated with aggravated inflammatory responses and a decreased number of immune cells, which provides a reference for the disease severity assessment and treatment of patients with sepsis.

## 1. Introduction

Sepsis is an infectious disease caused by multiple inflammatory factors that cooperate in the pathology of immune dysfunction and is the leading cause of death in intensive care unit (ICU) patients [1–3]. As a syndrome characterized by uncontrollable inflammation, sepsis involves a massive and persistent inflammatory response and a prolonged state of immunosuppression, leading to an increased risk of secondary nosocomial infections [4, 5]. Factors such as inflammatory response, immune response, and infection are risk factors for the development of sepsis, and the disease may be exacerbated by dysregulation of inflammatory factor levels in patients [3, 6]. Relevant studies have shown that the risk of sepsis infection in diabetic patients is 2.5-6.0 times higher than in nondiabetic patients. Moreover, patients with sepsis are predisposed to insulin resistance due to glycemic changes [5, 7]. Previous research has also demonstrated that the higher the blood glucose level in sepsis patients, the more severe the patient's condition [8]. The present study was conducted to explore the correlation between blood glucose levels and inflammation and immune indicators in patients with sepsis.

## 2. Materials and Methods

### 2.1. General Information

From February 2019 to February 2021, 30 sepsis patients and 30 sepsis patients complicated with diabetes mellitus admitted to our hospital were recruited and assigned to either the experimental group (sepsis patients) or the observation group (sepsis patients with diabetes mellitus). Another 30 healthy subjects in the same period were included in the control group. After enrollment, blood pressure was measured with an electronic manometer. The II acute physiology and chronic health evaluation were performed.

### 2.2. Inclusion Criteria and Exclusion Criteria

The following are the inclusion criteria: patients who met the diagnostic criteria for sepsis, those with sepsis and diabetes who met the 2018 version of the diagnostic criteria for diabetes issued by the American Diabetes Association (ADA), and those who provided written informed consent after being fully informed of the purpose and process of the study. This study was ratified by the hospital ethics committee.

The following are the exclusion criteria: patients with tumors, those who were pregnant or lactating women, those with autoimmune diseases, those with severe mental illnesses, and those with medications that might affect the blood glucose levels, such as glucocorticoids.

### 2.3. Method

After admission, 4 ml of venous blood was collected from all subjects. 2 ml of blood sample was naturally clotted at room temperature for 30 min, followed by centrifugation at 1000 g for 10 min to obtain the serum. The serum was then stored at -20°C for assays. A multiplex microsphere flow-through immunofluorescence luminescence was used to determine the levels of interleukin-6 (IL-6), tumor necrosis factor-*α* (TNF-*α*), and interleukin-1*β* (IL-1*β*). The remaining 2 ml of blood samples was anticoagulated with EDTA-K2 at room temperature, vortexed and mixed for 3 seconds, and used for the determination of the CD4+ and CD8+ levels with flow cytometry.

12 cytokine assay kits were purchased from Qingdao Risskell Biotechnology Co., Ltd., China; CD3+, CD4+, and CD8+ fluorescent monoclonal antibody kits were purchased from Beijing Tong Sheng Times Biotechnology Co.

### 2.4. Observational Indicators

The levels of IL-6, TNF-*α*, IL-1*β*, CD4+, and CD8+ in the three groups of patients were compared to analyze the correlation of blood glucose levels with inflammatory response and immune indicators in patients with sepsis.

### 2.5. Statistical Analyses

SPSS20.0 was used for data analyses, and GraphPad Prism 7 (GraphPad Software, San Diego, USA) was used to plot the graphics.The counting data are expressed as [*n*(%)]and analyzed using the chi-square test, and the measurement data are expressed as $\bar{x}\pm s$ and analyzed using the *t*-test. Statistically significant result was defined as *P* < 0.05.

## 3. Results

### 3.1. Baseline Patient Profile

There were no significant differences in the baseline characteristics between the three groups of participants (*P* > 0.05). See Table 1.

### 3.2. Comparison of IL-6 Levels of Inflammatory Factors in the Three Groups

**Figure 1:**
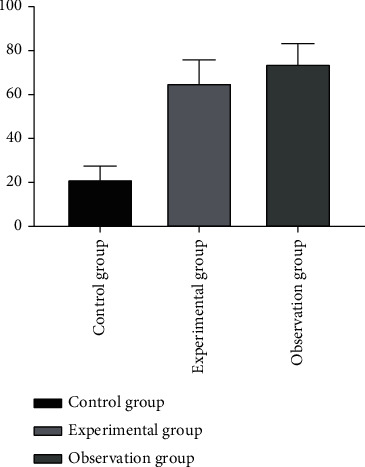
Comparison of IL-6 levels of inflammatory factors in the three groups ($\bar{x}\pm s$). Note: the abscissa represents the control group, experimental group, and observation group; the ordinate represents the level of IL-6 (pg/ml); the level of IL-6 in the control group was 14.32 ± 4.98 pg/ml; the level of IL-6 in the experimental group was 18.33 ± 3.27 pg/ml; the level of IL-6 in the observation group was 22.64 ± 5.16 pg/ml; ∗ indicates that there is a significant difference in IL-6 levels between the control group and the experimental group (*t* = 3.687, *P* < 0.001); ∗∗ indicates that there is a significant difference in IL-6 levels between the control group and the observation group (*t* = 6.355, *P* < 0.001); ∗∗∗ indicates that there is a significant difference in IL-6 levels between the experimental group and the observation group (*t* = 3.864, *P* < 0.001).

The levels of inflammatory factors including IL-6 were the lowest in the control group, followed by the experimental group, and then the observation group (*P* < 0.05). See Figure 1.

### 3.3. Comparison of TNF-*α* Levels of Inflammatory Factors in the Three Groups

**Figure 2:**
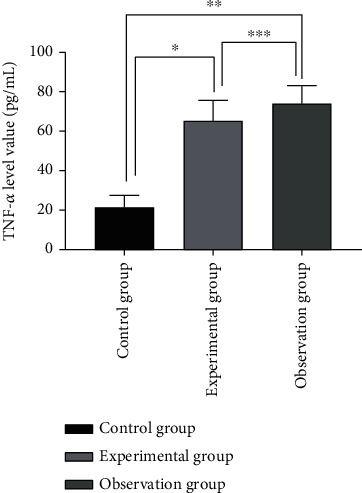
Comparison of TNF-*α* levels of inflammatory factors in the three groups ($\bar{x}\pm s$). Note: the abscissa represents the control group, experimental group, and observation group; the ordinate represents the level of TNF-*α* (pg/ml); the level of TNF-*α* in the control group was 21.42 ± 6.03 pg/ml; the level of TNF-*α* in the experimental group was 65.27 ± 10.32 pg/ml; the level of TNF-*α* in the observation group was 74.05 ± 9.14 pg/ml; ∗ indicates that there is a significant difference in the TNF-*α* level between the control group and the experimental group (*t* = 19.765, *P* < 0.001); ∗∗ indicates that there is a significant difference in the TNF-*α* level between the control group and the observation group (*t* = 26.326, *P* < 0.001); ∗∗∗ indicates that there is a significant difference in the TNF-*α* level between the experimental group and the observation group (*t* = 3.488, *P* < 0.001);

The level of TNF-*α* in the control group was significantly lower than that of the other two groups (P <0.05), and the observation group showed a higher level of TNF-*α* than the experimental group (*P* < 0.05). See Figure 2 for details.

### 3.4. Comparison of IL-1*β* Levels of Inflammatory Factors in the Three Groups

**Figure 3:**
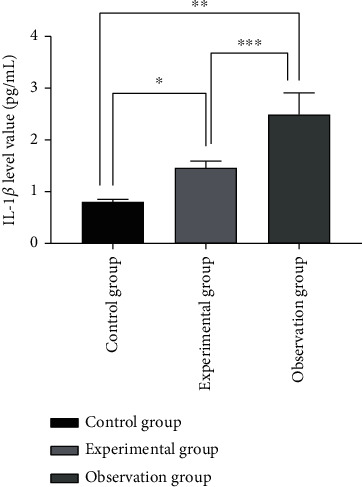
Comparison of IL-1*β* levels of inflammatory factors in the three groups ($\bar{x}\pm s$). Note: the abscissa represents the control group, experimental group, and observation group; the ordinate represents the level of IL-1*β* (pg/ml); the level of IL-1*β* of the control group was 0.80 ± 0.05 pg/ml; the level of IL-1*β* of the experimental group was 1.46 ± 0.13 pg/ml; the level of IL-1*β* in the observation group was 2.49 ± 0.42 pg/ml; ∗ indicates that there is a significant difference in IL-1*β* levels between the control group and the experimental group (*t* = 25.954, *P* < 0.001); ∗∗ indicates that there is a significant difference in IL-1*β* levels between the control group and the observation group (*t* = 21.885, *P* < 0.001); ∗∗∗ indicates that there is a significant difference in IL-1*β* levels between the experimental group and the observation group (*t* = 12.832, *P* < 0.001);

The levels of inflammatory factors including IL-1*β* were the lowest in the control group, followed by the experimental group, and then the observation group (*P* < 0.05). See Figure 3.

### 3.5. Immune function indicators

Sepsis patients with diabetes mellitus in the observation group were associated with the lowest CD4+ and CD8+ levels in the control group, followed by the sepsis patients in the experimental group, and then the healthy participants in the control group (*P* < 0.05). See Table 2.

### 3.6. Correlation Analysis of Blood Glucose Levels and IL-6, TNF-*α*, and IL-1*β* Levels in Patients with Sepsis

**Figure 4:**
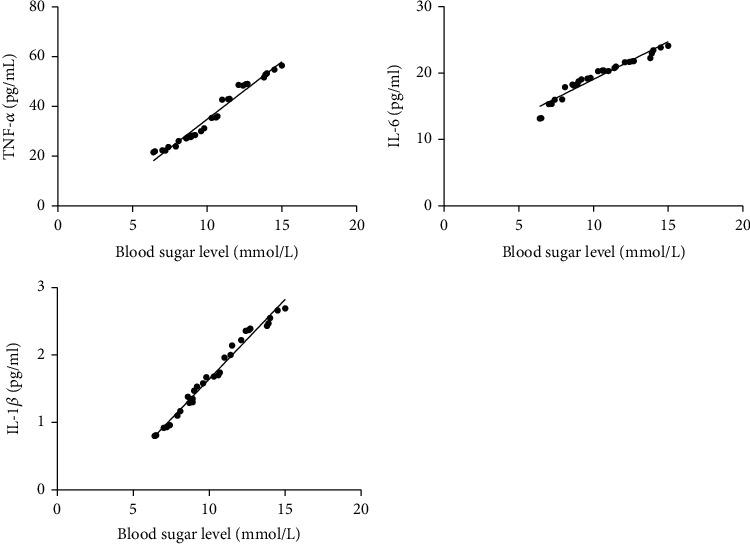
Correlation analysis of blood glucose levels and IL-6, TNF-*α*, and IL-1*β* levels in patients with sepsis. Note: the abscissa indicates the blood glucose level; the ordinate indicates the level of IL-6, TNF-*α*, and IL-1*β*.

The blood glucose level of patients with sepsis was positively correlated with the levels of IL-6, TNF-*α*, and IL-1*β*. The higher the blood glucose level, the higher the level of IL-6, TNF-*α*, and IL-1*β*. See Figure 4.

### 3.7. Correlation Analysis of Blood Glucose Levels and CD4+ and CD8+ Levels in Patients with Sepsis

**Figure 5:**
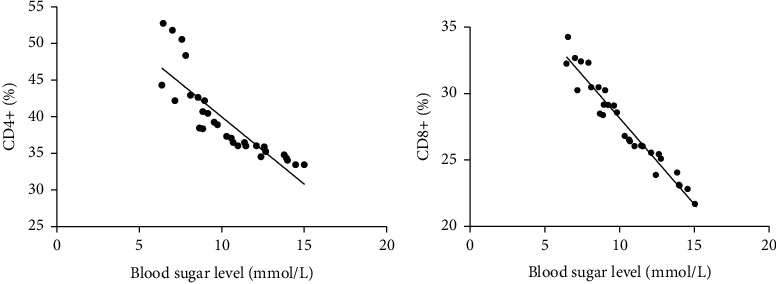
Correlation analysis of blood glucose levels and CD4+ and CD8+ levels in patients with sepsis. Note: the abscissa represents the blood glucose level; the ordinate represents the level of CD4+ and CD8+.

The blood glucose level of patients with sepsis was negatively correlated with the levels of CD4+/CD8+. The higher the blood glucose, the lower the number of immune cells. See Figure 5.

## 4. Discussion

Sepsis is a critical illness in intensive care medicine and is associated with protein, sugar, and lipid metabolism disorder and intensive inflammatory responses [9, 10]. Inflammation is a defensive measure of the body in the face of traumatic injury. Mitochondrial damage-related pattern molecules are released when the body is subject to strong stimuli such as severe trauma, burns, and major surgical operations, which initiate inflammatory responses through activation of neutrophils and mitogen-activated protein kinase signaling pathways [11–13]. IL-6 is a broad pleiotropic cytokine that plays a key role in host defense by regulating immunity and inflammatory responses [14]. TNF-*α* and IL-1*β* are proinflammatory factors that stimulate T cells to secrete large amounts of inflammatory mediators, which compromise the immune function and exacerbate the systemic inflammatory response [15–17]. CD4+ is an important immune cell that is primarily involved in infection control in the body. CD8+ is generated during the differentiation of lymphocyte surface antigens and can identify different stages of lymphocyte development. Studies have demonstrated that CD8+ belongs to an important subgroup of T lymphocytes and exerts cell proliferation and expansion effects after differentiation to directly participate in the body's immune regulation [18–20]. It has been found that there is an interplay between the blood glucose level of patients with sepsis and the development of the disease. Specifically, high blood sugar levels may aggravate inflammatory responses and impair the body's resistance, thereby accelerating disease development [21].

The levels of inflammatory factors including IL-6, TNF-*α* and IL-1*β*, were the lowest in the control group, followed by the experimental group, and then the observation group (*P* < 0.05), which is consistent with the research results by Kim et al. [22], indicating a severe systemic inflammatory response in patients with sepsis complicated with diabetes. In addition, a previous study showed that the serum IL-6 level was significantly positively correlated with blood glucose level upon intensive care unit (ICU) admission (*n* = 153; *r* = 0.24, *P* = 0.01) [23]. In the present study, the lowest immune function indicators CD4+ and CD8+ levels were found in the observation group, followed by the experimental group, and then the control group (*P* < 0.05), suggesting that patients with sepsis are associated with abnormal expression levels of T lymphocytes, which underscores the role of the assay of CD4+ and CD8+ levels for early diagnosis and treatment of sepsis. Moreover, the blood glucose level of patients with sepsis was positively correlated with the levels of IL-6, TNF-*α* and IL-1*β* and was negatively correlated with the levels of CD4+/CD8+; the higher the blood glucose, the lower the number of immune cells, which is in line with the results of the report by Nakamura et al. [24], indicating that the high blood glucose levels are associated with more severe immunity dysfunction in the patients with sepsis. As blood sugar levels increase, IL-6, TNF-*α* and IL-1*β* levels present a trend of increase, whereas the level of CD4+/CD8+ shows a downward trend. In conclusion, the blood glucose level of patients with sepsis is positively correlated with inflammatory response and negatively with immune indicators. An increased blood sugar level is associated with aggravated inflammatory responses and a decreased number of immune cells, which provides a reference for the disease severity assessment and treatment of patients with sepsis.

## Figures and Tables

**Table 1:** Comparison of clinical data of patients in the three groups [*n* (%)].

Items	Control group (*n* = 30)	Experimental group (*n* = 30)	Observation group (*n* = 30)	*χ* ^2^/*t*	*P*
Gender					
Male	14 (46.67)	13 (43.33)	11 (36.67)	0.6171	0.432
Female	16 (53.33)	17 (56.67)	19 (63.33)		
Average age (year)	45.62 ± 7.58	48.27 ± 8.13	46.33 ± 7.42	0.367	0.715
Systolic blood pressure/(kPa)	16.34 ± 4.28	17.10 ± 4.35	16.55 ± 4.29	0.19	0.85
Diastolic blood pressure/(kPa)	11.17 ± 2.64	10.89 ± 2.83	11.46 ± 2.70	0.421	0.676
APACHE*ΙΙ* scores	21.60 ± 6.57	24.55 ± 7.41	24.48 ± 6.63	1.69	0.096

**Table 2:** Comparison of immune function indicators in the three groups ($\bar{x}\pm s$).

Groups	*n*	CD4+ (%)	CD8+ (%)
Observation group	30	34.61 ± 10.25	22.28 ± 10.21
Experimental group	30	49.36 ± 9.34^∗^	29.94 ± 11.18^∗#^
Control group	30	58.54 ± 10.27^∗#^	35.46 ± 9.67^∗#^

∗ indicates compared with the control group, *P* < 0.05; # indicates compared with the experimental group, *P* < 0.05.

## Data Availability

The datasets used during the present study are available from the corresponding author upon reasonable request.
